# Flash-and-Freeze: Coordinating Optogenetic Stimulation with Rapid Freezing to Visualize Membrane Dynamics at Synapses with Millisecond Resolution

**DOI:** 10.3389/fnsyn.2016.00024

**Published:** 2016-08-19

**Authors:** Shigeki Watanabe

**Affiliations:** ^1^Department of Cell Biology, Johns Hopkins UniversityBaltimore, MD, USA; ^2^The Solomon H. Snyder Department of Neuroscience, Johns Hopkins UniversityBaltimore, MD, USA

**Keywords:** flash-and-freeze, synaptic transmission, synaptic cell biology, synaptic vesicle exocytosis, synaptic vesicles, optogenetics, high-pressure freezing, electron microscopy

## Abstract

Electron microscopy depicts subcellular structures at synapses exquisitely but only captures static images. To visualize membrane dynamics, we have developed a novel technique, called flash-and-freeze, which induces neuronal activity with a flash of light and captures the membrane dynamics by rapid freezing. For characterizing membrane movements during synaptic transmission, a light-sensitive cation channel, channelrhodopsin, is heterologously expressed in mouse hippocampal neurons or in *Caenorhabditis elegans* motor neurons. A brief pulse of blue light activates channelrhodopsin and induces an action potential, leading to synaptic transmission. Following the light stimulation, neurons are frozen at different time intervals ranging from 10 ms to 20 s. Electron micrographs are then acquired from each time point to visualize the morphological changes. Using this approach, we have characterized a novel form of endocytosis, ultrafast endocytosis, which rapidly removes excess membrane added to the surface during neurotransmission. The flash-and-freeze approach can be adapted to study other cellular phenomena that can be induced by light-sensitive genetic or pharmacological tools.

## Introduction

### Imaging neurons

To process information at synapses, membrane trafficking takes place on a millisecond time scale. When a nerve impulse arrives at synaptic terminals, synaptic vesicles fuse with the plasma membrane within 1 ms (Heuser and Reese, 1981). Subsequently, vesicle membrane and proteins are recovered from the plasma membrane to reconstitute new vesicles (Dittman and Ryan, 2009; Saheki and De Camilli, 2012). One major challenge in studying membrane dynamics at synapses is visualizing these rapid events at the spatial resolution necessary to resolve individual vesicles and temporal resolution relevant to synaptic functions. Several methods have been developed over the years including electrophysiological, optical, and electron microscopy methods.

Capacitance measurements of synaptic terminals allow monitoring of membrane flux at the plasma membrane with sub-millisecond temporal resolution (Hamill et al., 1981; Neher and Marty, 1982). The capacitance across a membrane is proportional to the membrane area. Direct patch-clamping of synaptic boutons shows an increase in the capacitance during exocytosis and a decrease during endocytosis (von Gersdorff and Matthews, 1994; Sun et al., 2002; Delvendahl et al., 2016). This method has a single vesicle sensitivity. However, it is blind to the locations of the membrane insertion and removal. Furthermore, membrane trafficking within the cytosol cannot be visualized by this technique.

Optical imaging methods aim to label functional pools of vesicles in the terminals and visualize their fates. The spatial resolution of optical microscopy is limited to ~200 nm by the diffraction of light. Because synaptic vesicles are small (~40 nm in diameter) and hundreds of these vesicles are clustered in a confined space, optical microscopy cannot resolve individual vesicles in the terminal. Therefore, several tricks have been developed to visualize specific subsets of vesicles. For example, fluid phase markers like dextran, ferritin, and quantum dots can be applied exogenously to label endocytosed vesicles and visualize their dynamics in the terminal (Zhang et al., 2007b; Park et al., 2012). Similarly, lipophilic dyes like FM dyes and mCling can be internalized via endocytosis and mark the newly reconstituted vesicles (Betz et al., 1992; Revelo et al., 2014). Finally, pH-sensitive fluorescent molecules such as pHluorin (Miesenböck et al., 1998) can be used to monitor synaptic vesicle cycle. Fluorescence from pHlourin is quenched in the lumen of synaptic vesicles due to the low pH. When exposed to the extracellular space by exocytosis, the protein becomes fluorescent. The pHluorin molecules are quenched again once they are internalized and vesicles are fully re-acidified. These techniques allow visualization of particular vesicles (Sankaranarayanan and Ryan, 2000; Balaji and Ryan, 2007; Armbruster et al., 2013). However, like in the capacitance measurement, it is difficult to visualize intracellular trafficking events, limiting the interpretation of the results.

Electron microscopy, on the other hand, depicts all the membrane-bound structures within the terminal, but only captures a static image. To overcome this limitation, neurons are typically stimulated and fixed at defined time points after the stimulation (Ceccarelli et al., 1972; Heuser and Reese, 1973; Ferguson et al., 2007; Clayton et al., 2008; Matthews and Sterling, 2008; Hoopmann et al., 2010; Schikorski, 2014; Wu et al., 2014). However, the temporal resolution is limited to seconds to minutes for two reasons. First, to ensure the capture of endocytic events, neurons are often stimulated with prolonged intense stimulation that lasts seconds to minutes. Second, diffusion and reaction of aldehyde-based chemicals are slow, requiring an additional time. To visualize membrane dynamics on a millisecond time scale, Heuser and Reese developed the “freeze slammer,” which freezes tissues near instantaneously following electrical stimulation (Heuser et al., 1979; Heuser and Reese, 1981). Using this device, they were able to observe vesicles fusing with the plasma membrane 5 ms after an electrical pulse (Heuser and Reese, 1981; Heuser et al., 1979). Although individual images are static, membrane dynamics can be visualized in electron micrographs by inducing a particular activity and freezing neurons at multiple defined time points.

The freeze slamming approach allows visualization of membrane dynamics at the temporal resolution relevant to synaptic functions, but it has two major drawbacks. First, the specimen must be physically attached to the electrical wire, and thus experiments can only be performed *ex vivo*. Second, only the surface of specimens (~5 μm) can be frozen without ice crystal damage. These two factors limit its utility as a versatile tool for studying membrane trafficking at synapses. To overcome these limitations, we have developed a novel technique, flash-and-freeze, that combines optogenetics with high-pressure freezing (Watanabe et al., 2013a,b, 2014a). With this technique, non-invasive light stimulation is used to induce synaptic transmission. Specimens as thick as 200 μm can be properly frozen without ice crystal formation. Therefore, intact animals like *Caenorhabditis elegans* (*C. elegans* hereafter) or an entire population of neurons in a dish can be studied. Here, I will describe the methods in detail and discuss its potential applications in synaptic cell biology.

### High-pressure freezing

An electron microscope operates under high vacuum to avoid the scattering of electrons by gaseous molecules in the air. Thus, to observe a specimen in a transmission electron microscope, it must be fixed and dehydrated. In addition, electron beam must penetrate through the specimen, requiring extremely flat specimens with a thickness of ~30–70 nm. For this reason, the specimen is embedded in plastic and sectioned ultrathin. The sample preparation for electron microscopy often leads to the generation of artifacts. Fixation using aldehyde-based chemicals cross-links proteins and aggregates them. Worse yet, this reaction can induce fusion of synaptic vesicles (Smith and Reese, 1980). Furthermore, dehydration leads to the shrinkage of the membrane-bound structures and the overall changes in the morphological architecture of the cells. Therefore, a better approach must be used to study membrane trafficking events at synapses.

One approach to avoid these artifacts is to immobilize cells physically by rapid freezing. The freezing process, however, leads to formation of ice crystals that can damage the cellular architecture by directly penetrating through the membrane. Alternatively, the solutes segregated from ice crystals can burst membrane due to the local changes in the osmotic pressure. To prevent water molecules from forming ice crystals, a freezing rate of at least 10,000 K/s must be achieved. At this rate, water molecules cannot rearrange to form ice crystals and are frozen in an unordered state. The cooling rate by liquid nitrogen can exceed 16,000 K/s. Unfortunately, the heat conductance of water is poor, reducing the rate to 1000 K/s within 10 μm from the point of the contact. Under high pressure (2100 bar), however, a freezing rate of 100 K/s is sufficient to freeze water in an unordered state due to the supercooling effect (Moor, 1987; Dubochet, 2007). Thus, high-pressure freezing allows freezing of tissues up to 200 μm thickness or intact animals like *C. elegans*, (~70 μm thick) without ice crystal damage.

### Optogenetics

To visualize membrane dynamics during neurotransmission, neurons must be stimulated and frozen at defined time points after stimulation. In the freeze slammer, specimens are physically attached to the stimulating wire, limiting the specimen choice. Furthermore, the configuration of the high-pressure freezer makes it difficult to apply electrical field stimulation. To overcome these limitations, we have coupled optogenetics with high-pressure freezing. Channelrhodopsin is a light-sensitive cation channel isolated from *Chlamydomonas reinhardtii* (Nagel et al., 2003). A flash of blue light opens the channel, allowing cations to flow into the cell, thereby depolarizing it. When the channel is heterologously expressed in neurons, a short pulse of light triggers an action potential, leading to synaptic transmission (Boyden et al., 2005; Nagel et al., 2005). Therefore, non-invasive stimulation can be applied to a population of neurons in a dish or intact animals.

To couple optogenetic stimulation with high-pressure freezing (“flash-and-freeze”), we have developed a device that interfaces with the computer of high-pressure freezer as well as with an LED (Watanabe et al., 2013a,b, 2014a). This device allows application of light pulses at defined time points before the specimen is frozen (see Section Materials and Methods). Using this approach, we can visualize the membrane trafficking events at synapses with a millisecond temporal resolution.

## Materials and methods

**Figure 1:**
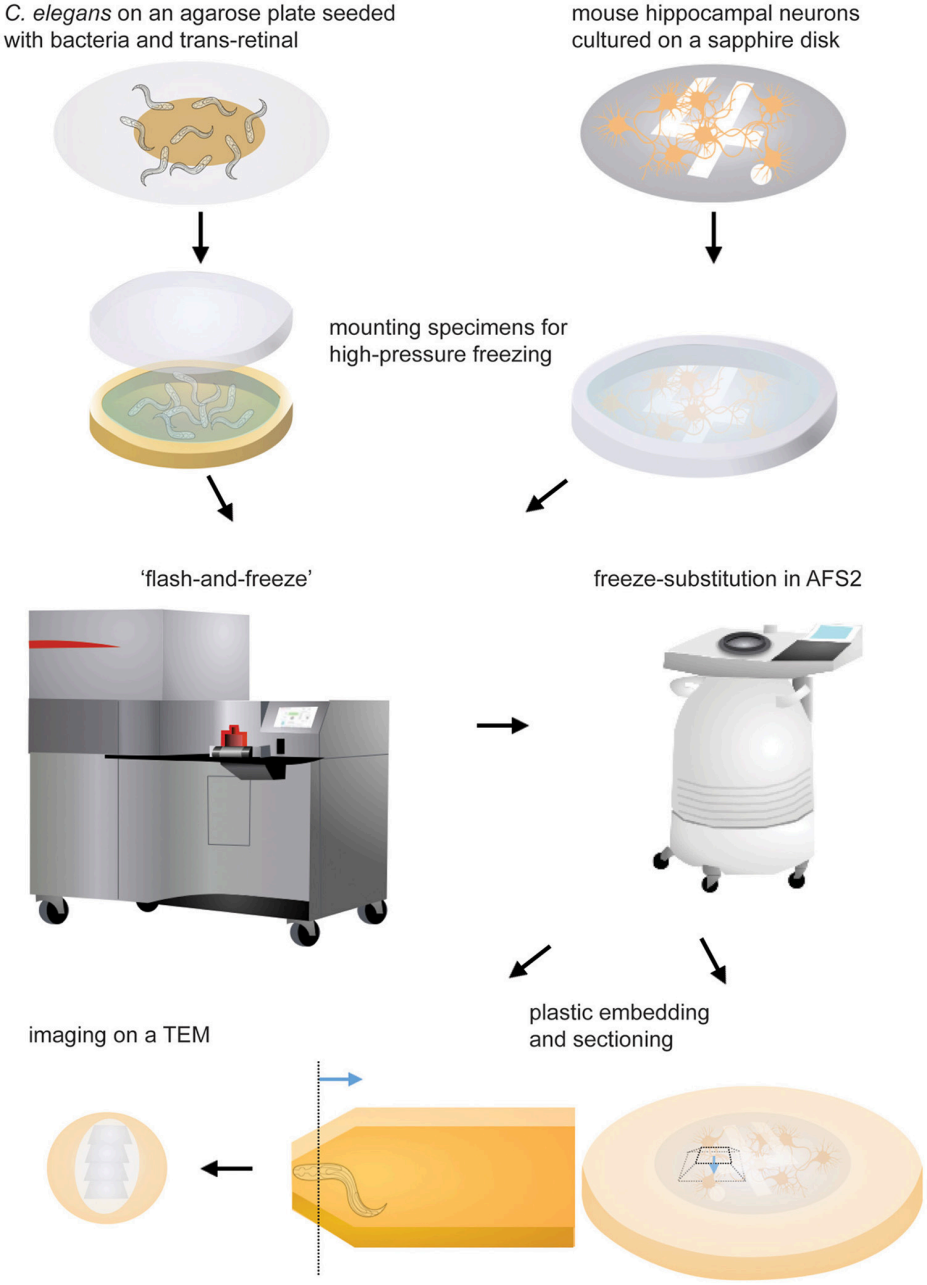
**Schematic drawings showing the experimental procedures**. Specimens are mounted in the appropriate carrier and frozen immediately following light stimulation. The freeze-substition is carried out in an automated freeze-substitution unit. Once the freeze-substitution is complete, specimens are embedded in resin and cured for 48 h. The region of interest (the dotted line) is located using a stereoscope. The blue line indicates the direction of sectioning. The ultrathin sections are mounted on a grid and imaged on a transmission electron microscope.

All experiments are performed according to the guidelines for the animal use by the National Institute of Health. The animal protocol is approved by the Animal Care and Use Committee at Johns Hopkins University, School of Medicine. The graphical representations of the workflow is shown in Figure 1. The step-by-step protocol can be found in the Supplementary information.

### Cell cultures

For flash-and-freeze experiments, specimens must be mounted on a substrate that is translucent like a glass coverslip but can withstand the extreme pressure application. Sapphire disks are ideal substrates, meeting these two criteria. In addition, thermal conductivity of sapphire is extremely high, particularly at low temperature, making it the perfect substrate. For culturing cells on sapphire disks, the following procedures are performed. First, we sputter carbon on one side of 6 mm sapphire disks so that we can distinguish the surface that the cells are cultured on. Then, we scratch out a letter “4” on the carbon-coated surface with a diamond scribe. Alternatively, a finder grid can be placed on a sapphire disk as a mask. The sapphire disks are baked at 120°C overnight. After a brief dip in ethanol, two sapphire disks are placed in each well of a 12-well plate and air-dried in a biosafety cabinet. Poly-D-Lysine solution (acetic acid 3 parts, rat tail collagen 1 part, and poly-D-lysine 1 part) is applied directly on each sapphire disk. After 5 min, the excess solution is removed, and the plates are dried under the laminar flow. Prior to use, the 12-well plates containing sapphire disks are sterilized by exposure to UV light for 20 min.

For hippocampal cultures, we first culture astrocytes as a feeder layer and then plate the neurons on top of the astrocytes. Mouse brains are dissected out from newborn C57/BL6J mice immediately after decapitation. Cortices and hippocampi are isolated from each brain, in cold HBSS solution. For the astrocyte feeder layer, cortices are treated with 0.05% Trypsin-EDTA for 20 min at 37°C. The dissociated astrocytes are then cultured in DMEM containing 10% FBS and 0.1% penicillin-streptomycin for 2 weeks in a T-75 flask. Then astrocytes are transferred to the 12-well plate containing sapphire disks (5 × 10^4^/ well) and grown for a week. Astrocyte mitosis is arrested a few hours before neuron plating using fluorodeoxyuridine (80 mm). Media are exchanged with Neurobasal, a media containing 2% B27, 1% glutamax, and 0.1% penicillin-streptomycin, prior to neuron plating. Isolated hippocampi are treated with papain (20 U/ ml) for 1 h and plated (5 × 10^4^/well) on top of the feeder layer. Neurons are allowed to grow for 2–3 weeks. On DIV1-3, neurons are infected with lentivirus containing Channelrhodopsin expressed from the neuron specific synapsin promoter.

###  *Caenorhabditis elegans*

Transgenic animals expressing ChIEF, a variant of channelrhodopsin-2, are grown on an agar plate (50 mm) seeded with 250 μl of *E. coli* OP50. Larval stage 4 (L4) transgenic animals are transferred to agar plates seeded with bacteria and 4 μl trans-retinal solution (100 mM) a day prior to the experiment. These animals are kept in the dark before the experiments.

### Freezing

**Figure 2:**
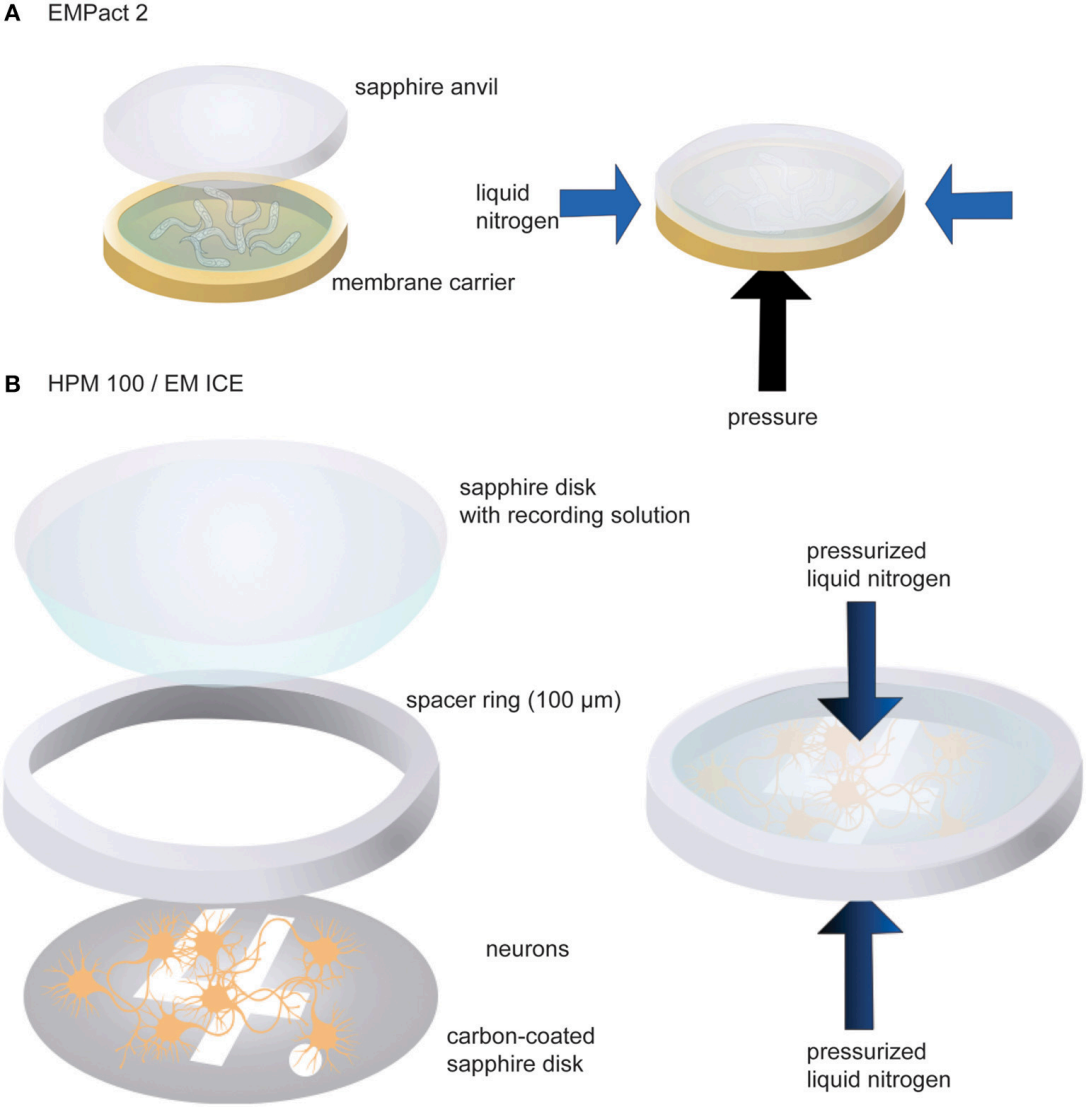
**Schematic drawings showing sample loading in the EM PACT2 (A) and in the HPM 100 or the EM ICE (B)**. For the EM PACT2, the membrane carrier must be sealed with a sapphire anvil before the experiments. Typically, the liquid is slightly overfilled so that no air bubbles are trapped when sealing with the sapphire anvil. For the HPM 100 and the EM ICE, liquid should be carried with the top sapphire disk to seal the assembly with no bubbles. For the EM PACT2, the pressure is applied from the bottom of the membrane carrier while liquid nitrogen is applied from the side of the specimen. For the HPM 100 and the EM ICE, pressurized liquid nitrogen is applied to the specimen from both sides.

We have used three different high-pressure freezers: Leica EM PACT2, HPM100, and ICE. *C. elegans* has been tested in the EM PACT2, whereas neuronal cultures are frozen with the HPM100 or ICE. For the EM PACT2, a membrane carrier with a 100 μm-deep well is used to mount transgenic worms (Figure 2A). First, the well of the membrane carrier is filled with M9 worm buffer containing 20% bovine serum albumin (BSA) as cryo-protectant. Approximately 10–15 young adult animals are transferred into the solution. The membrane carrier is then mounted into the modified specimen pod.

To mount cultured neuronal cells, the following procedures are carried out (Figure 2B). First, a sapphire disk with neurons is transferred into pre-warmed (37°C) recording solution containing 140 mM NaCl, 2.4 mM KCl, 10 mM HEPES (pH 7.5), 10 mM glucose, 4 mM CaCl_2_, 1 mM MgCl_2_, 3 μM NBQX, and 30 μM bicuculline. The addition of NBQX and bicuculline blocks the recurrent network activity following channelrhodopsin stimulation. The sapphire disk is mounted on the countersink of a 6-mm middle plate. A spacer ring (100 μm thickness) is placed on the sapphire disk so that cells are not crushed when the assembly is capped with another sapphire disk. To cap the assembly, a sapphire disk is first dipped in the recording solution so that one side of the disk carries a drop of solution. This sapphire disk is then placed on top of the spacer ring gently so that air bubbles are not trapped in between the disks. Two more spacer rings (200 μm each) are then placed to fill up the remaining space. The excess solution should be removed by gently tapping with a small piece of filter paper. The middle plate assembly is then sandwiched between two transparent plastic half cylinders. Note that light is shone from the bottom side of the specimen assembly in the HPM100 while it is from the top in the ICE. The video demonstrating sample loading can be found on the Leica website (http://www.leica-microsystems.com/science-lab/video-tutorials-filling-and-assembling-of-different-carriers-for-high-pressure-freezing/).

### Programming light stimulation

The modifications we have made for the EM PACT2 are described in detail in previous publications (Watanabe et al., 2013a,b, 2014a). For controlling the light stimulation and freezing, we constructed an Arudino Uno based device that sends out 5V TTL signals to the LED and the high-pressure freezer at the desired time point. This device is driven by custom firmware to produce any pattern of light pulses (i.e., single stimulus, 10 Hz stimulation) and send out the “start” signal that triggers the freezing process (this device can be purchased from Marine Reef International). Once the signal is sent, the freezer applies hydraulic fluid to the specimen and pressurize the specimen. When the pressure reaches 2000 bar, the liquid nitrogen is immediately applied to the specimen. The initiation of this freezing process needs to be timed so that the desired time interval between the light stimulation and freezing is achieved. To monitor the precise timing when the pressure is applied to the specimen, we installed an accelerometer on the sample holder (also known as a “specimen bayonet”). When the pressure is applied to the specimen, the sample holder jolts, producing a distinctive peak in the accelerometer recording. We found that it requires about 170 ms for the EM PACT2 to apply the pressure to the specimen after sending the ‘start’ signal. After the pressure is applied to the specimen, the specimen is frozen to −20°C in 8 ms, according to standard thermodynamic equations. To account for the variability in timing, we calculate the actual time interval *post-hoc* based on the pressure peak recorded by the accelerometer. We have found the actual time interval to typically be within 33.6 ± 4.6 ms of the intended time.

To introduce light stimulation capability in the HPM100, a similar Arudino Uno based device was constructed. The operation of the HPM100 is fundamentally different from that of the EM PACT2 in that pressurized liquid nitrogen is applied to the specimen instead of a hydraulic fluid. After sending the “start” signal, it takes 370 ms to compress the liquid nitrogen. The pressurized liquid nitrogen reaches the specimen in precisely 72 ms. The specimen is frozen to −20°C in 8 ms from the time the pressurized liquid nitrogen hits the specimen surface. Therefore, a total of 450 ms delay is expected. To monitor the precise timing, light stimulation device record the internal signal of the machine that opens the liquid nitrogen valve in each shot. This signal initiates almost invariably at 370 ms after the start signal. The actual timing of the freeze is adjusted *post-hoc* based on the valve opening signal.

For EM ICE, Leica microsystems has integrated the light stimulation control into the freezer. The machine is capable of freezing specimen at the desired timing after the light stimulation with 1–2 ms variability.

### Freeze-substitution

Following high-pressure freezing, vitrified water from the specimen must be substituted with organic solvent to avoid crystallization of vitrified water as the specimen warms up to room temperature for further processing. The freeze-substitution is carried out in cryotubes containing fixatives (1% osmium tetroxide, 1% glutaraldehyde, 1% milliQ water in anhydrous acetone) using an automated freeze-substitution unit (Leica AFS2). The cryotubes containing fixatives are stored under liquid nitrogen to avoid the cross-reaction between osmium tetroxide and glutaraldehyde. The sapphire disks containing the cells typically remain associated with the middle plate. To release the sapphire disks, the middle plate is quickly transferred to a cup containing acetone, which is precooled to −90°C in AFS. Once the middle plate reaches −90°C in the acetone, the sapphire disk often dissociates from the middle plate spontaneously. If not, it can be released by a gentle tap. The sapphire disk is then transferred into the cryotube containing the fixatives at −90°C. For *C. elegans*, the frozen specimens can be transferred under liquid nitrogen into the cryotubes containing fixatives. The tubes should be transferred quickly into the AFS. The following program is used for the freeze-substitution: −90°C for 5–30 h, 5°C/h to −20°C, 12 h at −20°C, 10°C/h to 20°C. The cryotubes are agitated at least twice a day during the substitution process.

### Plastic embedding

Once the freeze-substitution is complete, specimens are embedded in Epon/Araldite resin. The resin is as composed of 4.4 g Araldite 502, 6.2 g Eponate 12, 12.2 g dodecenyl succinic anhydrite (DDSA), and 0.8 ml benzyldimethylamine (BDMA). First, the fixative solution is carefully removed from the cryotube, and specimens are washed with acetone a few times. Specimens are then treated with 0.1% uranyl acetate in acetone for 1 h to enhance the membrane contrast. Following several acetone washes, infiltration is carried out in the same cryotubes on an orbital shaker: 30% for 2–5 h, 70% for 3–6 h, and 90% overnight at room temperature. The next day, specimens are transferred into the caps of BEEM capsules containing 100% Epon/Araldite resin. The resin is replaced three times (2 h each). The specimens are cured in an oven (60°C) for 48 h.

### Sectioning

For observation in a transmission electron microscope, specimens must be sliced thin so that electrons can penetrate through the tissue and generate an image. Ultrathin sections (40 nm) of specimens are cut using a diamond knife and collected on pioloform-coated single slot grids. For *C. elegans*, we orient the animal so that it is perpendicular to the sectioning surface (Figure 1 bottom). We trim the animal to the reflex of the gonad using a glass knife. We collect ribbons of 250 sections from each animal. For mouse hippocampal neurons, we first remove the sapphire disks from the sample by submerging them in the liquid nitrogen for a few seconds. About 40 sections are cut from the exposed surface (Figure 1 bottom) and collected. These sections are stained with 2.5% uranyl acetate in 70% methanol for 4 min prior to imaging.

### Imaging

A transmission electron microscope is operated at 80–120 keV. Images are acquired with a digital camera. Roughly 200–300 images are collected from each time point.

### Image analysis

The morphological analysis is performed in ImageJ using a custom-written macro. This macro records X- and Y-coordinates of hand-traced membrane structures such as the plasma membrane, active zone, membrane invaginations, synaptic vesicles, dense-core vesicles, large vesicles, and endosomes. The positional information is first exported as text files and then imported into MATLAB (MathWorks). We have written scripts in MATLAB to analyze the coordinates and calculate several statistics: distribution of vesicles from the plasma membrane, distribution of vesicles from the active zone, diameter of vesicles, and number of vesicles. A normality test is performed on these data, and *P*-values are calculated using student's *T*-test for normally distributed data and Mann–Whitney *U*-test for skewed data. The confidence level is set at 95%. For multiple comparisons, the Bonferroni correction is applied.

## Results

### Accuracy of timing

To test the accuracy of the timing for light stimulation, we applied a single stimulus and froze tissues at desired time points. We calculated the actual time at which the specimens were frozen by monitoring the time when the pressure was applied to the specimens. The pressure application can be monitored using an accelerometer for EM PACT 2 (Watanabe et al., 2013b, 2014a). For HPM 100, we recorded the signal that triggers opening of the valve that allows pressurized liquid nitrogen to pass through because specimens are frozen exactly 80 ms after the valve opening (see Section Materials and Methods; Watanabe et al., 2013a). On the EM ICE, we can measure the interval between the end of the desired stimulation program and the time at which the sensor, which is located close to the specimen, reaches 0°C. The sensor is placed before the specimen, and it requires additional 3-6 ms for the specimen to experience the same magnitude of cooling.

**Figure 3:**
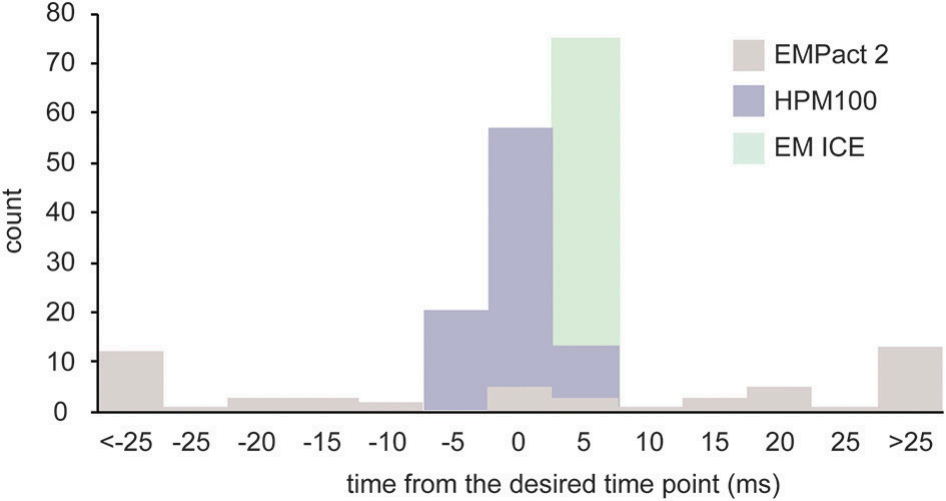
**A bar graph showing the time difference between the desired time point and the actual time point at which specimens are frozen**. The variation is largest in the EM PACT2, with the average delay of 33.6 ± 4.6 ms (*N* = 4 experiments; *n* = 52 shots). The average delay of the HPM100 is 1.9 ± 0.3 ms (*N* = 3 experiments; *n* = 90 shots) while that of the EM ICE is 2.2 ± 0.1 (*N* = 3 experiments; *n* = 72 shots).

We recorded the values from each machine on at least three different experimental days. Out of 52 shots taken on the EM PACT2 (4 experiments, 13 shots/experiment), the desired time point was achieved in only 5 shots (Figure 3). The actual timing was variable, ranging from −102 to 110 ms. Because of this variability, the actual timing is always calculated *post-hoc* for each shot on the EM PACT2. On the HPM100, nearly two thirds of the shots (57/90) achieved the desired time point (3 experiments, 30 shots/experiment). The rest were either 5 ms before or after the desired time point, indicating that the variation is small in the HPM 100. However, the signal for the valve opening is measured at 5 ms time interval (200 Hz), and thus each recording has an uncertainty of ±2.5 ms. The EM ICE, on the other hand, records the temperature and pressure sensors' signals at 2000 Hz. The data from the EM ICE indicated that 56 out of 72 shots (3 experiments, 24 shots/experiment) landed between 1.5 and 2.5 ms after the desired time point. The maximum delay was 4 ms, and this maximum delay occurred on the very first shot on each experimental day. The rest of the shots were within 1.5–3.5 ms after the desired time point (average = 2.2 ± 0.08 ms). Given that the additional 3 ms is required to freeze specimens at 5 μm deep from the surface, a total of 4.5–6.5 ms is expected. These results indicate that millisecond temporal control can be achieved in HPM 100 and EM ICE.

### Exocytosis

**Figure 4:**
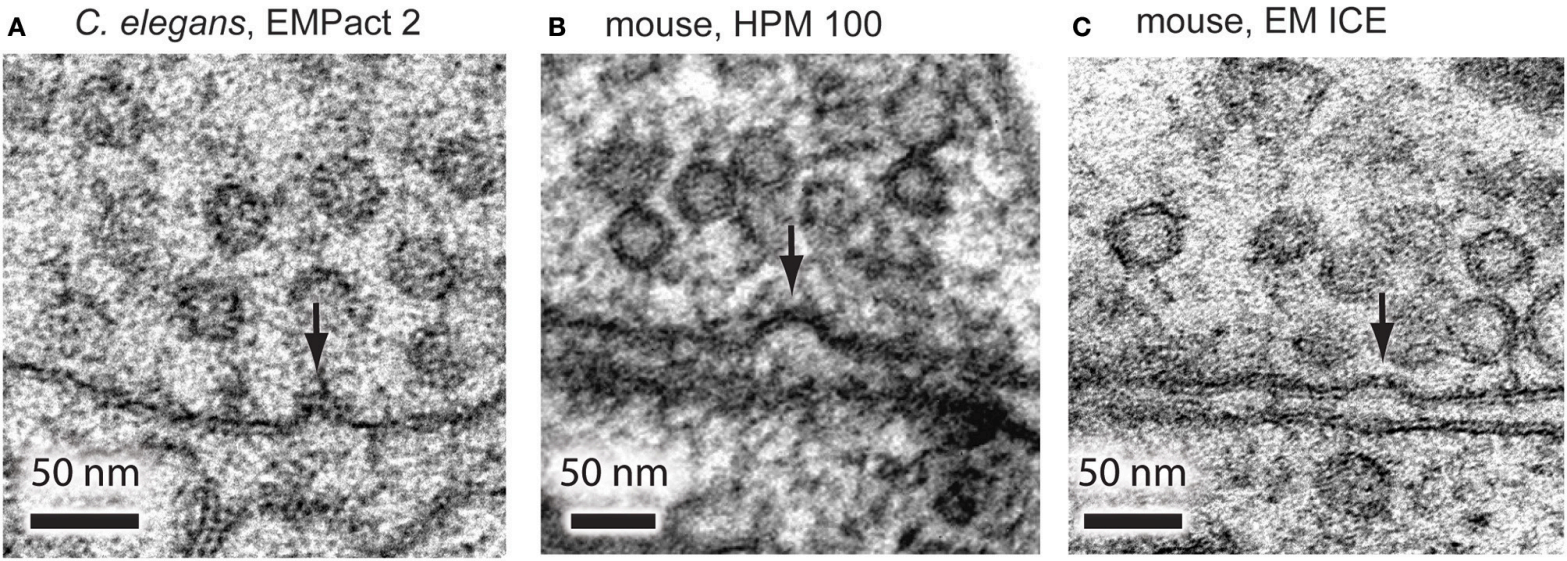
**Representative micrographs showing exocytic omega figures from the flash-and-freeze experiments. (A)** *C. elegans* neuromuscular junctions, stimulated for 20 ms, and frozen at the end of the light pulse. Mouse hippocampal neurons, stimulated for 10 ms, frozen 5 ms after the end of the pulse using HPM 100 **(B)**, and EM ICE **(C)**. These structures are rarely observed in non-stimulated control or in specimens frozen at later time points after the stimulation.

To further test the accuracy of the timing, we stimulated *C. elegans* neuromuscular junctions for 20 ms and froze them at the end of the pulse using the EM PACT2. Based on the electrophysiological recordings, channelrhodopsin induced neurotransmission is close to the peak at this time point in the *C. elegans* motor neurons (Watanabe et al., 2013b). From a total of 13 shots taken, only 1 shot achieved this time point. To further test the accuracy, we also imaged the samples that were frozen 10 ms before the onset of the light pulse and 30 ms after the end of the light pulse, as calculated *post-hoc*. We were able to capture exocytic omega figures at neuromuscular junctions frozen 20 ms after the light onset (Figure 4A). However, these structures were not observed in the samples frozen 10 ms before the light onset or 30 ms after the end of the light pulse. These results indicate that the EM PACT2, although unreliable, can be used to capture fast membrane dynamics at synaptic terminals.

To test if the exocytic omega figures can be captured with the HPM 100 and the EM ICE, we froze mouse hippocampal neurons expressing channelrhodopsin. We applied a single 10 ms light pulse to the specimen and froze it 5 ms after the end of the light pulse. In the neurons frozen on the HPM 100, we found at least one omega figure in 19% of synapses at this time point (Figure 4B; 193 profiles; Watanabe et al., 2013a). Similarly, omega figures were captured in 18% of synapses by EM ICE (Figure 4C; 105 profiles). These results indicate that the flash-and-freeze approach can reliably capture the membrane dynamics at synapses with milliseconds temporal resolution.

## Discussion

### Comparisons among three instruments

To visualize membrane dynamics at synapses, we developed a technique, “flash-and-freeze,” which couples optogenetic stimulation of neurons with high-pressure freezing. Here, we compared three instruments that allow such experiments: EM PACT2, HPM100, and EM ICE. There are advantages and disadvantages for each instrument. The major advantage of the EM PACT 2 is in its potential application - higher intensity of ultraviolet light can be applied to specimens because light only has to penetrate through a clear sapphire anvil. Most caged compounds are uncaged with ultraviolet light. In both HPM100 and EM ICE, light must travel through an optical fiber and penetrate through a plastic sample holder that absorbs ultraviolet light. Thus, experiments using caged compound are more feasible in the EM PACT2. However, the EM PACT2 required many trials before the desired time point was reached. Besides the unreliability, the major disadvantage of the EM PACT2 is the size of the specimen cup. A specimen cup has a dimension of 1.6 mm diameter × 100 or 200 μm depth. Cells must be cultured on sapphire disks with a diameter of 1.4 mm. These small sapphire disks float in culture media, making it difficult to culture cells. On the other hand, sapphire disks as large as 6 mm can be loaded into the HPM100 or the EM ICE. Both the HPM100 and the EM ICE are essentially comparable in terms of the freezing quality and the temporal precision of light stimulation. However, HPM100 is more flexible in terms of its potential applications, as the light stimulation device and programs are customizable. With the expansion in the repertoire of optogenetic tools or light-sensitive compounds, cellular activity can be controlled using different wavelengths of light, and it is possible to develop a multi-color system for the HPM100. The major disadvantages of HPM 100 are that it requires an external device and that the actual freezing point must be calculated based on when the liquid nitrogen valve opens each time. On the contrary EM ICE does not require a custom device or an external light source—everything is integrated. Furthermore, it achieves the most precise control of the light stimulation and the fastest freezing rate, requiring only 3 ms from the initial temperature drop to −20°C. Despite these differences among instruments, exocytosis of synaptic vesicles, which occurs on a millisecond time scale, were captured in all three instruments, suggesting that any of these machines are compatible with flash-and-freeze experiments. Therefore, the choice should be made depending on the potential applications.

### Potential applications

The flash-and-freeze approach can be applied to study many cell biological problems. We have been using channelrhodopsin to stimulate neuronal activity and capture membrane dynamics at synapses. Neuronal activity or cellular functions can be manipulated using light-sensitive molecules such as bacterial opsins (Han and Boyden, 2007; Zhang et al., 2007a; Berthold et al., 2008; Chow et al., 2010), caged compounds (Walker et al., 1986; Tsien et al., 1987; Milburn et al., 1989; Adams and Tsien, 1993; Wieboldt et al., 1994), light-sensitive proteins (Levskaya et al., 2009; Wu et al., 2009; Yazawa et al., 2009; Idevall-Hagren et al., 2012; Zhou et al., 2012; Guntas et al., 2015), and photo-isomerizable molecule (Banghart et al., 2004; Kramer et al., 2009). These tools can be used to inhibit or activate neuronal activity, protein function, lipid composition, signaling cascades, and ion composition in vesicular structures. The morphological changes that take place during these manipulations can be preserved through the flash-and-freeze approach and visualized with a millisecond temporal resolution. Furthermore, there are neurons like photoreceptors that are naturally sensitive to light. The flash-and-freeze approach can be readily adapted to address the mechanism underlying tonic release of neurotransmitters from these terminals and how exocytosis is mediated by synaptic ribbons, electron dense proteinaceous protrusion in the center of the active zone (LoGiudice and Matthews, 2009).

### Potential difficulties

Membrane dynamics are inferred by observing the membrane morphology at defined time points, but individual electron micrographs still capture static images of cells. The flash-and-freeze technique cannot follow the membrane dynamics in a single cell. Therefore, to reconstruct the membrane dynamics, hundreds of images must be acquired and analyzed from each time point. For membrane trafficking at synapses, an even higher number may be required due to the heterogeneity in the release probability (Rosenmund and Stevens, 1996)—not every action potential leads to fusion of synaptic vesicles. We have estimated that about 20–30% of synapses are activated with a single light pulse in mouse hippocampal neurons (Watanabe et al., 2013a, 2014b). To produce data with statistical significance, we have analyzed over 10,000 images. These analyses were performed blind to genotypes and time points, and thus the average frequency of morphological features at a particular time point can be determined from these images. To expedite analysis and increase the statistical power, automation is in an immediate need. However, with the recent advances in methods for automated image acquisition and analysis (Potter et al., 1999; Denk and Horstmann, 2004; Knott et al., 2008; Hayworth et al., 2014), achieving such a goal is likely within reach.

## Author contributions

SW performed all the experiments, analyzed the data, and wrote the manuscript.

### Conflict of interest statement

The author declares that the research was conducted in the absence of any commercial or financial relationships that could be construed as a potential conflict of interest.
