# Supplementary material for: Flash-and-Freeze: Coordinating Optogenetic Stimulation with Rapid Freezing to Visualize Membrane Dynamics at Synapses with Millisecond Resolution
Source: Front Synaptic Neurosci. 2016 Aug 19;8:24. doi: 10.3389/fnsyn.2016.00024 (PMC4990539; doi:10.3389/fnsyn.2016.00024)
Supplement: Supplementary file 1 [file DataSheet1.PDF]

Watanabe, Supplementary information

The step-by-step experimental procedures are described below.

Preparation of specimens

Mouse hippocampal neurons

1. Apply carbon coating on sapphire disks (6 mm; Technotrade #616-100). Using a diamond scribe, draw a character such as “4” on the surface so that the surface on which cells are cultured can be easily distinguished from the other surface.
2. Following a brief wash in ethanol, place the sapphire disks in 12-well plates, and apply poly-D-lysine solution (acetic acid 3 parts, rat tail collagen 1 part, and poly-D-lysine 1 part) for 5 min. Remove the excess solution and air dry under the laminar flow cabinet. Prior to seeding, apply UV light for 20 min.
3. Dissociate astrocyte from cortices by treating them with 0.05% Trypsin-EDTA for 20 min at 37 ºC. Incubate astrocytes in DMEM media containing 10% FBS and 0.1% penicillin-streptomycin for two weeks in a T-75 flask. Note that this procedure should be conducted three weeks prior to culturing neurons. Seed the astrocytes on the 12-well plate containing sapphire disks at a density of $5 \times 10^4$/ well. Let them grow at 37 ºC for 1 week.
4. Stop the mitosis of astrocyte using fluorodeoxyuridine (80 uM) 3-4 hours before neuron seeding.
5. Dissociate hippocampal neurons from P0-P2 pups using papain (20 U/ ml) for 1 hour and plate them ($5 \times 10^4$ /well) on top of the feeder layer.
6. Using lentivirus, transfect neurons with the expression construct carrying ChetaTC, a variant of Channelrhodopsin, at days *in vitro* (DIV) 1-3.
7. Perform flash-and-freeze experiments at the desired age (typically DIV14-21).

*C. elegans* motor neurons

1. Raise *C. elegans* transgenic animals expressing ChIEF, a variant of channelrhodopsin, in the dark.
2. Transfer L4 animals to agar plates seeded with 250 µl of *E. coli* OP50 and 4µl trans-retinal solution (100 mM). Keep them in dark until the experiments on the next day.

Preparation of freeze-substitution media

1. Prepare the freeze-substitution media by mixing 0.25 g osmium tetroxide, 2.5 ml glutaraldehyde (10% stock in acetone: EMS), 250 µl milliQ water in 50 ml conical tube and top it off to 25 ml with anhydrous acetone. Note that glutaraldehyde and osmium teteroxide cross-react at room temperature, and thus, the solution must be aliquoted and frozen in liquid nitrogen as quickly as possible.
2. Aliquot ~1 ml to cryotubes. Mark the cryotubes with a pencil. Submerge the cryotubes into liquid nitrogen, and keep them under liquid nitrogen until further use.

High pressure freezing

1. The specimens are mounted on the high-pressure freezer as shown in Fig. 1. Hippocampal neurons are mounted with the pre-warmed (37ºC) recording solution containing 140 mM NaCl, 2.4 mM KCl, 10 mM HEPES (pH 7.5), 10 mM glucose, 4 mM CaCl2, 1 mM MgCl2, 3µM

NBQX, and 30 μM bicuculline. *C. elegans* can be mounted with M9 containing 20% BSA or *E. coli* OP50.
2. Keep the specimen in dark for 15-30 s to let it recover from any potential stimulation by ambient light.
3. Apply light stimulation and freeze specimens at the desired time points. Following the freeze, the specimens must be kept under liquid nitrogen all the time until further processing.

Freeze-substitution
1. Under the liquid nitrogen bath, transfer the sample into the cryotubes containing freeze-substitution media. For hippocampal cultures, the sapphire disk must be dissociated in acetone at -90ºC. Transfer the middle plate containing the frozen specimen into a cup filled with precooled acetone in the automated freeze substitution unit (Leica AFS2). Gently tap out the sapphire disks from the middle plate, and transfer the sapphire disk into the cryotube containing the media.
2. Run the following program in the AFS: 5-30 hours at -90ºC, 5ºC/hour to -20ºC, 12-16 hours at -20ºC, 10ºC/hour to 20ºC. Agitate the cryovials at least twice a day.

*en bloc* staining (optional)
1. After freeze-substitution, wash off the media with anhydrous acetone by aspirating off the media slowly and pipetting in acetone. Repeat the procedures 3-6 times to wash off the media completely.
2. After the final wash, pipet in ~1 ml acetone containing 0.1% uranyl acetate.

Plastic embedding
1. After the acetone wash, infiltrate specimens with plastic (araldite 4.4 g, epon 6.2 g, DDSA 12.2 g, and BDMA 0.8 ml): 30% for 3-5 hours, 70% for 4-6 hours, 90% overnight.
2. On the next day, prepare fresh resin. Place specimens in an appropriate mold. Apply 100% resin into the mold. Replace the media twice every two hours.
3. After the final exchange, cure the resin in a 60ºC oven for 48 hours.

Sectioning
1. For *C. elegans*, orient the animal so that they will be perpendicular to the knife edge. For hippocampal cultures, remove the sapphire disk by dipping the resin in liquid nitrogen for a few seconds and prying the disk up with forceps. Using a glass knife, cut the specimen to the appropriate location. For *C. elegans* motor neurons, we trim down the animals to the anterior reflex of gonad. For hippocampal neurons, we slice off the first 300 nm, which mainly contains astrocytes.
2. Collect the desired number of sections using an ultramicrotome. We typically collect 250 sections (33 nm thick) for *C. elegans* neuromuscular junctions and 40 sections (40 nm thick) for hippocampal cultures. The ribbons of sections are collected onto TEM slot grids coated with 0.5-0.7% pioloform.

Imaging
1. Acquire images on a transmission electron microscope. For statistical purposes, we typically acquire ~200 images from each time point. The image acquisition is carried out blind to the treatment or genotypes. The subsequent image analysis is also blinded.
